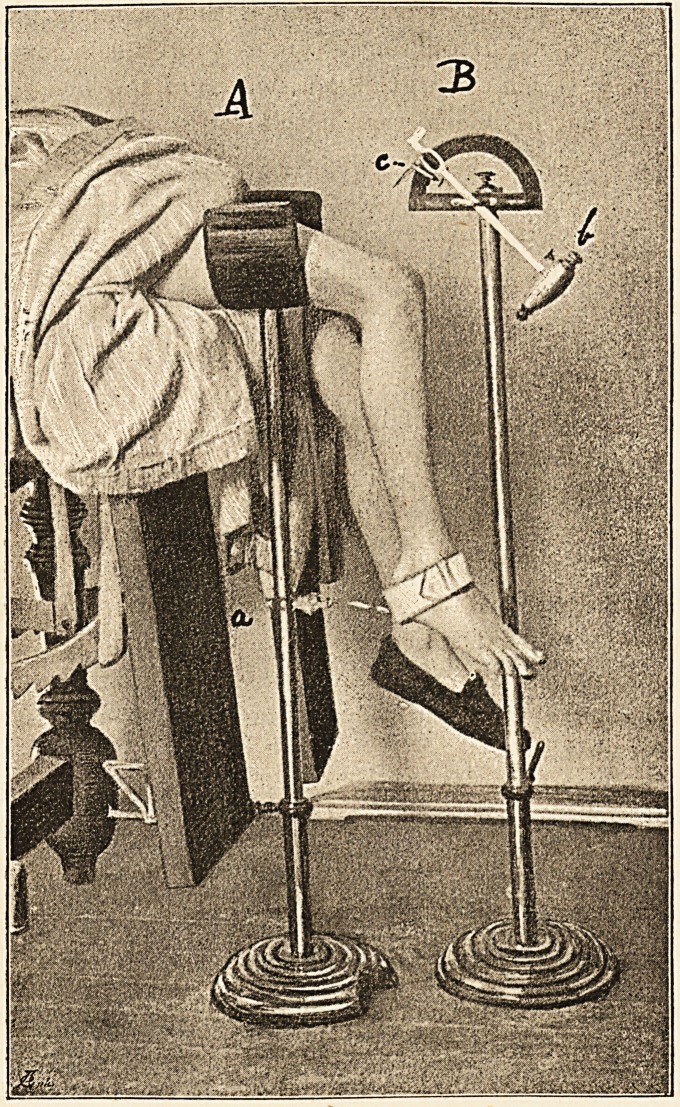# The Quantitative Estimation of the Knee-Jerk

**Published:** 1892-03

**Authors:** Ewen J. Maclean

**Affiliations:** House Surgeon Bristol Hospital for Sick Children and Women


### THE QUANTITATIVE ESTIMATION OF THE KNEE-JERK.

BY

EWEN J. MACLEAN, M.D., C.M. Edin.,

*House Surgeon Bristol Hospital for Sick Children and Women.*

THE state of the knee-jerk, as reflecting amongst other things, and often very graphically, the functional condition (to speak generally) of the spinal cord, would appear to be worthy of very close and accurate clinical observation.

Comparison of the writings of the leading classical authors on nervous diseases shows that there is often much disparity, amounting at times to absolute contradiction in statement, as to the condition of the knee-jerk. An inherent individual variability must ever be reckoned upon, as accounting for some amount of disparity; but after the theoretical elimination of this, there still remain some such causes as the following:

1. Non-mention of the stage of the disease to which the recorded state of knee-jerk corresponds.
2. Faulty position of patient's body during elicitation of the jerk.
3. Faulty position of limb.
4. Varying strength of percussion.
5. Varying point of impact on patellar tendon.
6. Varying ocular judgment of jerk amplitude.

I believe that the instrument which is the subject of this article, whilst eliminating many variation factors, affords a ready clinical means of testing for the presence or absence of the knee-jerk, and when the jerk is present, its character, including its amplitude and, if necessary, time-interval.

The instrument[1] (see illustration), which may be termed a "Jactometer," is composed of two main parts:

**A** A limb-rest, with amplitude registration apparatus (*a*).

**B** Percussion stand.

**A** The limb-rest consists of an upright fixed in a wooden foot-piece, which has a crescentic portion removed, so that in the case of a very short limb being tested no inconvenience is occasioned by the contact of the foot-piece of **B**.

[1] Messrs. Down Bros., of St. Thomas's Street, London, S.E., exercised much care in carrying out my design.

A
B
c
b
a

The upright is so constructed as to be adaptable, in major degrees, by the unscrewing and removal of one of its component rods; in minor degrees, by a telescopic sliding arrangement with hand-screw, to any convenient height requisite for the thigh, which rests on a wide padded trough terminating the limb-rest.

The quantitative registration of any particular jerk is effected by means of a graduated (English and metric systems) tape, which being fixed at one end to the foot by means of a padded strap, is free to unroll itself, by a very slight pull, from off the tape-roll (*a*), which, having a lateral spring for the purpose, readily allows the tape to run out when tension is applied by the projecting foot, but does not unroll any tape by its own acquired momentum; so that two desiderata are thus secured; (1) the least possible amount of resistance is offered to the projecting foot, and (2) no fallacious amplitude is contributed. The tape-roll is so attached to the upright that, by sliding it upwards or downwards, any convenient level required by the position of the foot may be obtained.

**B** The percussion stand.

The foot-piece and adaptable upright, as in **A**. At a right angle to the upper extremity of the upright is placed a graduated arc, which by means of a sliding joint and screw in the head of the upright is horizontally adaptable, so that percussion of the tendon at its inner, middle, or outer part may be secured at will.

The percussion is effected by means of a lever hammer (*b*) pivoted at the centre of the base of the arc. The head of the hammer is lower, somewhat torpedo-shaped, tipped with a layer of india-rubber, and weighs 13 oz. Five ounces of this are removable by the screw and central weight arrangement. By this device the relation of the amplitude to the momentum of impact may be estimated. The upper end of the hammer projects beyond the arc, and is bent at a right angle so as to come in contact with a Marey tambour (not shown in illustration), fixed at a convenient point of the arc, for the time-interval estimation. In order to deliver a stroke, the upper end of the lever is drawn towards the patient and fixed at the required angle on the arc, where a small horizontal pin attached to it is held by a tip-catch (*c*). Slight pressure on that end of the tip-catch nearer the patient liberates the hammer, which falls, delivering a stroke varying in momentum with the amount of weight of its head and the angle at which its upper end was held, and varying in point of percussion with the position of the horizontal joint above referred to: other things being equal.

The apparatus is obviously equally adaptable to either limb.

The observation is taken with the patient preferably (though not necessarily) in the supine position and at rest; the limb to be tested rests easily and horizontally over the limb-rest, which has been previously adapted to the required height, the centre of the trough being at about the middle of the thigh; thus any pressure on the sciatic nerve or posterior part of thigh generally is obviated, and the limb is in the best possible position for elicitation of the knee-jerk. The leg hangs freely and comfortably. The foot-strap is then adjusted and connected with the tape and the tape-roll, which is brought to the level of the heel. The observer next adjusts the percussion stand, and taking a seat facing the patient, and with one foot resting on the foot-piece in order to steady it and prevent vibration from any unevenness of the floor, he brings the tip of the hammer to that point of the tendon he wishes to be struck, and secures it opposite this position by tightening the screw at the sliding point; then bringing back the hammer-head, and fixing the upper end by the catch at the required angle on the arc and regulating at pleasure the amount of weight to be used, he presses the catch, the hammer falls, percusses the tendon at the required spot, the foot flies forward (if the jerk is present), and the extent to which it does so is recorded on the tape in the English and metric systems. And this is the knee-jerk amplitude.

The time-interval, the apparatus for which is not shown in the illustration, may also be estimated with a Marey's tambour fixed at a point of the arc where the bent upper end of the hammer comes into contact with the drum exactly at the time when the india-rubber tip touches the tendon firmly. This may be readily so adapted. Another tambour may be placed in contact with the end of the upper arm of a lever fixed, at a convenient level, to the upright of the limb-rest; the lower end of this lever must be arranged so as to be raised by the very slightest tension on a very light chain attached to the heel-strap on the foot, and, by means of an arrangement provided for the purpose, due care must be taken to keep the chain taut; the tambour tubes are led away to their respective needles, and register on a revolving cylinder the moment of impact and that of first movement of the foot. As the tubes are of equal length the loss of time is equal. A chronographic tracing, taken by means of a reed vibrating 100 per second, gives the time value of the interval. A third tambour from the thigh will give the time of the first appreciable contraction of the quadriceps, and thus valuable time-comparisons are obtainable.

By the courtesy of Dr. Ferrier and other members of the staff of the National Hospital for the Paralysed and Epileptic, a number of nervous cases have been examined in detail. Considerations of interest respecting the results of percussion of the various parts of the patellar tendon, and of utilising the various methods of reinforcement, have been made out, as well as some deductions affecting diagnosis and prognosis; but these are for the present reserved till further corroboration may have rendered them trustworthy.

## Figures and Tables

**Figure f1:**